# Placebo-related improvement with methylphenidate treatment in children with ADHD

**DOI:** 10.1007/s00787-024-02550-3

**Published:** 2024-08-10

**Authors:** Karen Vertessen, Jaap Oosterlaan, Pierre Bet, Marco Bottelier, Reino Stoffelsen, James M. Swanson, Annemiek Wisse, Jos Twisk, Marjolein Luman

**Affiliations:** 1 VU Amsterdam, Van der Boechorststraat 7, 1081 BT Amsterdam, The Netherlands; 2 University Psychiatric Centre, Katholieke Universiteit Leuven, Leuven, Belgium; 3 Department of Pediatrics, Emma Children’s Hospital, Amsterdam UMC Location University of Amsterdam, Amsterdam, The Netherlands; 4 Amsterdam Reproduction and Development Research Institute, Amsterdam, The Netherlands; 5 Department of Clinical Pharmacology and Pharmacy, Amsterdam UMC, VU Medical Center, Amsterdam, The Netherlands; 6 Child Study Center Accare, UMC Groningen, Groningen, The Netherlands; 7 Levvel Specialists in Youth and Family Care, Amsterdam, The Netherlands; 8 Department of Pediatrics, University of California, Irvine, USA; 9 ADHD Centraal, Rotterdam, The Netherlands; 10 Department of Epidemiology and Biostatistics, VU Medical Center, Amsterdam, The Netherlands

**Keywords:** Attention Deficit Disorder with Hyperactivity, Methylphenidate, Child, Central Nervous System Stimulants, Placebo

## Abstract

Non-specific effects of methylphenidate treatment, including expectancy and regression to the mean effects, contribute to the overall effect of methylphenidate on attention-deficit/hyperactivity disorder (ADHD) symptoms. Knowledge on the extent to which non-specific effects contribute to the overall effect and whether regression to the mean explains part of the non-specific effects, is currently lacking. A double-blind, randomized, placebo-controlled, cross-over trial was used to compare parent and teacher ratings of child ADHD symptoms at baseline and during treatment with placebo and 5, 10, 15 and 20 mg of methylphenidate, twice daily. Participants were 5-13-year-old children with a DSM-5 diagnosis of ADHD (N = 45). The extent to which non-specific effects contributed to the effects of methylphenidate was determined by ADHD symptom reductions observed with placebo versus reductions observed with active doses of methylphenidate. The influence of regression to the mean was examined by estimating the contribution of baseline ADHD symptom severity to the effects observed with placebo treatment. Data were analyzed using multilevel analyses. We observed significant non-specific effects of methylphenidate for parent-rated ADHD symptoms, but not for teacher-rated symptoms. For parent reported hyperactive/impulsive symptoms, higher baseline symptoms predicted larger effects with placebo, indicating regression to the mean effects. For parent-reports, a significant part of the overall effect of methylphenidate treatment is explained by non-specific effects. Our findings stress the importance of taking non-specific effects into account when evaluating methylphenidate treatment, by including teacher-reports and using a double baseline assessment during titration. Comparing active medication with a placebo in the titration trial has the potential to identify non-specific effects.

**Supplementary Information:**

The online version contains supplementary material available at 10.1007/s00787-024-02550-3.

## Introduction

Attention-deficit/hyperactivity disorder (ADHD) is one of the most frequently diagnosed childhood-onset psychiatric disorders [1] and is associated with significant impairments in several functional domains and reduced quality of life [2, 3]. Methylphenidate is recommended as a first line pharmacological treatment for ADHD in children and has been shown highly efficacious in reducing ADHD symptoms compared to treatment with placebo, with large effect sizes close to 1.0 [4].

While the pharmacological effects of methylphenidate on ADHD symptoms are relatively clear [4], thus far the clinical field has given little attention to the contribution of non-specific effects of methylphenidate on ADHD symptoms. In accordance with recent expert opinion [7–10], non-specific effects are defined as the amalgam of responses that cannot be attributed to methylphenidate (the specific pharmacological agent) and can be observed by using placebo treatment (treatment that appears similar, but without the pharmacological agent). In the past few years, research interest into non-specific effects has grown rapidly. It has been argued that these effects may contribute to a large extent to the success of pharmacological treatment, sometimes even more so than the pharmacological effect itself. Indicating that when the overall effects of methylphenidate are interpreted both pharmacological and non-specific effects [5–8] should be considered.

**Fig. 1:**
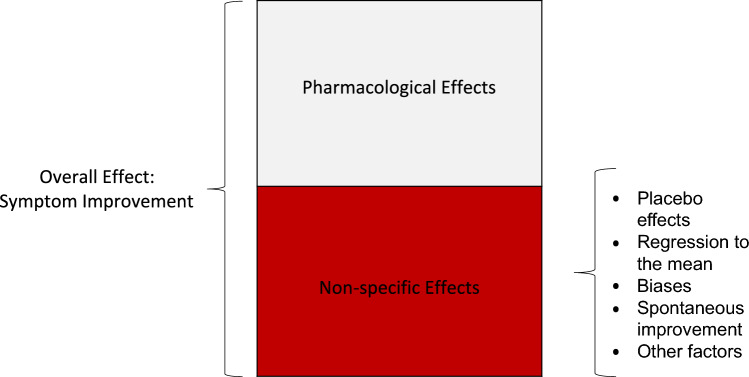
Overall, Pharmacological and Non-specific Effects of Methylphenidate Treatment Note: Overall Effect = symptom improvement compared to baseline; Pharmacological Effects = symptom improvement with medication compared to placebo treatment; Non-specific Effects: symptom improvement with placebo treatment compared to baseline. Visualization is based on the visualizations by Enck and Zipfel^7^ and Benedetti.^9^

In many papers the reduction of symptoms in a group that receives placebo treatment has been referred to as placebo response or placebo effect. However, this reduction in symptoms may also be explained by effects that are not related to actual placebo effect [10]. Placebo effects refer to the changes specifically attributable to placebo mechanisms, including the neurobiological and psychological mechanisms of expectancies [10]. Other factors that may contribute to improvement with placebo treatment, and are no part of the placebo effect, include spontaneous improvement, patient and /or observer bias and regression to the mean (please see Fig. 1) [10]. Our analysis focus on (a) the contribution of non-specific effects to clinically observed overall effects of methylphenidate and (b) the contribution of the regression to the mean to these non-specific effects. Regression to the mean is a statistical phenomenon that occurs when repeated measurements are made on the same subject or unit of observation [11]. Typically, individuals enrolled in clinical trials tend to display particularly high symptom levels (e.g., ADHD symptoms), i.e., higher than the individual’s long-term average, or "true" value, and these extreme values tend to be lower in subsequent measurements, this phenomenon is referred to as regression to the mean [11, 12].

A recent meta-analysis by Faraone and colleagues [6] showed significant improvement of ADHD symptoms under placebo treatment compared to baseline (i.e., measuring non-specific effects) in controlled ADHD medication trials. Effect sizes ranged between SMD 0.36 to 0.75 depending on the type of rater (e.g., parents, teachers, physicians) showing significant heterogeneity. The results imply that non-specific effects of methylphenidate can be differently perceived by different raters and the authors suggest taking rater-effects into account when examining improvement under placebo treatment [6].

In the meta-analysis by Faraone and colleagues [6] it was also observed that higher parent and/or teacher-rated severity of ADHD symptoms at baseline was associated to more improvement under placebo treatment. Several other studies, not included in that meta-analysis, have confirmed high levels of ADHD symptoms at baseline to predict improvement under placebo treatment [6, 13] and to be associated to greater symptom improvement with methylphenidate [14, 15]. A clear understanding of these findings is lacking. Regression to the mean may be an important contributing factor to the reported symptom decrease with placebo treatment (non-specific effects) [6], however, this has not been explored so far.

Taken together the current body of research clearly indicates that non-specific effects contribute to the overall effects of methylphenidate treatment on ADHD symptoms, however, (a) the extent to which non-specific effects contribute to the overall effect of methylphenidate and (b) the extent to which regression to the mean contributes to these non-specific effects is currently lacking.

The aim of the present study was twofold. First, we aimed to investigate the ratio of non-specific effects (placebo treatment versus baseline) compared to the overall effect of methylphenidate (active methylphenidate treatment versus baseline) using a double-blind, randomized, placebo-controlled, cross-over trial (PCT) in which children with ADHD were treated with placebo and different doses of methylphenidate. We used both parent and teacher ratings to assess ADHD symptom changes and thus explored rater-effects. We hypothesized that there would be significant improvement under placebo treatment, implying that non-specific effects play a role in the clinical perceived overall effect of methylphenidate [6]. Second, we aimed to investigate the contribution of regression to the mean effects to these non-specific effects by studying the impact of baseline ADHD symptoms on symptom improvement (separately for parent and teacher ratings) in the placebo treatment condition compared to baseline. We hypothesized that, if regression to the mean is an important aspect of non-specific effects, baseline symptom severity would positively predict symptom improvement [11].

## Method

The current study was part of a larger study into optimizing methylphenidate use in children with ADHD, comparing titration of methylphenidate as usual to PCT [16, 17], that was approved by the local ethics committee (METC Amsterdam UMC, # 2016.594) and registered prospectively in the Dutch trial register (# NL8121). All data of children participating in the PCT group (N = 45) were used in the current study.

### Sample

Children were recruited from mental health clinics in The Netherlands between May 2017 and December 2019. Inclusion criteria were: (a) a clinical diagnosis of ADHD according to DSM-5, (b) 5–13 years of age, (c) IQ > 70, (d) indication for methylphenidate treatment, as determined by the treating physician, and (e) no pharmacological treatment for ADHD at least four weeks prior to study entry. Comorbid diagnoses were not an exclusion criterion. The ADHD diagnosis was confirmed by the first author (K.V.) using the (1) Kiddie–Schedule for Affective Disorders and Schizophrenia for School-Age Children–Present and Lifetime Version (K-SADS-PL), a semi-structured standardized, investigator-based parent interview [18] and (2) teacher ratings on the Disruptive Behavior Disorder rating scale (DBDRS) assessing the pervasiveness and severity of symptoms of ADHD [19].

### Design

In order to investigate the pharmacological and non-specific effects of methylphenidate treatment, placebo and different doses of methylphenidate were administered to children using a double-blind, randomized, placebo-controlled, cross-over trial (PCT). The PCT protocol was based on the titration protocol used in the MTA study [20] and modified to improve clinical usability by: (1) adding a lead-in phase to determine if all doses used during titration were tolerated in terms of side effects, (2) weekly instead of daily dose changes to increase feasibility [21, 22], and (3) the use of an online tool to assess treatment outcomes. The titration procedure started with a lead-in phase, consisting of four days in which three (5, 10, 15 mg) or four (20 mg in children > 25 kg, limiting the maximum dose to 1 mg/kg/day) [21] doses of methylphenidate were administered in ascending order to assess tolerably. If a dose was not tolerated in the lead-in phase, it was excluded from the PCT. After lead-in, PCT started in which, in random order, placebo and each of the tolerated doses were administered for seven consecutive days, with the restriction that the highest dose was never the starting dose or the dose administered after placebo. This was done to reduce possible side effects due to sudden large dose augmentation. Duration of the PCT was three to five weeks, depending on the child’s weight and methylphenidate doses tolerated. During PCT, treatment with a particular dose started on a Saturday and was administered twice daily, at breakfast (around 8 a.m.) and at lunch time (around 12 a.m.).

### ADHD symptoms

ADHD symptom severity was measured with the Strengths and Weaknesses of Attention-Deficit/Hyperactivity Disorder Symptoms and Normal Behavior scale (SWAN) [23] completed by parents and teachers at baseline and for placebo and all dosages of tolerated methylphenidate. The SWAN is an 18-item rating scale measuring the presence and severity of ADHD symptoms on a continuum (from strength to difficulty). Items are rated on a 7-point Likert scale from − 3 (far below average) to + 3 (far above average) with lower scores indicating more severe ADHD symptoms. The SWAN usually requires informants to base their ratings on observations of the child’s behavior during the past month. For the current study, ratings pertained to the past week. Parent and teacher ratings on the Hyperactive/Impulsive and Inattentive scales were used separately as outcome measures. The SWAN has shown high internal reliability (0.94–0.96) and validity [23, 24]. The SWAN was sent out automatically to both parents and teachers through a tailor-made application build into an existing, and clinically widely used, online tool [25].

### Treatment expectations

To explore the role of treatment expectations custom-made questionnaires, described in the Supplement, were used to assess ‘Agreement with the diagnosis and therapy’ (combined score of four questions using a 5-point Likert scale) and ‘Treatment expectations’ (one question, using a 5-point Likert scale), with higher scores indicating higher agreement and higher expectations. A custom-made child questionnaire was used to assess the child’s ‘Opinion on diagnosis and treatment’ therapy’ (combined score of three questions using a 3-point Likert scale) and ‘Aversion towards medication’ (one question, using a 3-point Likert scale), higher scores indicating a more negative opinion towards diagnosis and treatment and a higher Aversion towards medication.

### Statistical analyses

Analyses were performed using STATA (version 17.0). First, dose was treated as a categorical variable, comparing the effects of placebo treatment versus baseline (measuring non-specific effects) to the effects of methylphenidate versus baseline (non-specific + pharmacological effects) on each of the four outcomes: parent and teacher rated hyperactive/impulsive symptoms and inattention symptoms. Symptom improvement was graphically displayed for all doses and multilevel analyses (mixed model analysis) were used to determine if placebo and 5, 10, 15, and 20 mg methylphenidate treatment led to significant improvement compared to baseline. For these analyses four hierarchical levels were distinguished: observations (Level 1), nested within children (Level 2), nested in physicians (Level 3), and nested in centers (Level 4). This nesting structure allows for the examination of how variables at each level (e.g., individual symptoms, child characteristics, physician factors, and clinic influences) contribute to the outcomes of interest. By accounting for these nested relationships, multilevel analyses enable us to model the variability within and between levels, providing a comprehensive understanding of the factors influencing our study outcomes. All multilevel analyses were adjusted for age and sex as lower age and male sex are related to higher baseline symptoms [2]. Random intercepts at physician and center level were only included if significantly improving model fit as determined by Likelihood Ratio Test. Second, for those outcomes that showed a significant improvement with placebo treatment (i.e., showing non-specific effects), we tested whether regression to the mean contributed to the effects. To this end we tested the relationship between severity of ADHD symptoms as assessed at baseline and the improvement in ADHD symptoms with placebo treatment, by adding an interaction term including the continuous variable baseline symptoms and the categorical variable methylphenidate dose to the multilevel models. Methylphenidate dose (placebo and 5, 10, 15 and 20 mg) was used to differentiate the effects of placebo from the active methylphenidate doses, but for these analyses we only report on effects of placebo treatment compared to baseline (i.e., assessing non-specific effects). Third, for those outcomes that showed a significant improvement with placebo treatment (i.e., showing non-specific effects), we tested whether treatment expectancies (from the person reporting symptom improvement and the child) contributed to the effects. To this end we tested the relationship between the variables for treatment expectations as assessed at baseline and the improvement in ADHD symptoms with placebo treatment, by adding an interaction term including the continuous variable and the categorical variable methylphenidate dose to the multilevel models. Methylphenidate dose (placebo and 5, 10, 15 and 20 mg) was used to differentiate the effects of placebo from the active methylphenidate doses, but for these analyses we only report on effects of placebo treatment compared to baseline (i.e., assessing non-specific effects).

### Procedure

Clinicians of the participating clinics informed parents and children on the study both verbally and through an information folder. If interested in participating, the first author provided parents and children with additional (written) information on the study. Parents and children older than 11 years provided signed informed consent. The physician delivered a prescription to the academic pharmacy, where randomization took place. Thereafter parents received the study medication required for the entire titration period and baseline assessment was conducted during that week. Demographic information and outcomes were assessed through questionnaires administered via the online tool. Participating clinics, clinicians and families involved in the study did not receive any reimbursement for their participation. Parents were instructed to contact the treating physician in case of severe side effects or other problems. These contacts were not aimed to provide specific coaching on behavioral problems. However, since the titration method was implemented in clinical care, limited guidelines or restrictions were provided to the participating physicians. This allowed physicians some discretion in addressing issues raised by parents, although the primary focus remained on monitoring side effects.

## Results

### Sample

Forty-one clinicians from 13 youth mental health clinics across the Netherlands recruited children for the larger study. One hundred and eight children were assessed for eligibility, of which 100 children fulfilled inclusion criteria. A total of 45 children was randomized to the PCT group and contributed data to the current study. Table 1 displays the participants’ demographic and clinical characteristics. One child did not receive the 15 and 20 mg due to a combination of severe side effects (rated by parents as troublesome) and dosing restriction (< 25 kg), eight children did not receive the 20 mg dose, six because of the dosing restrictions (< 25 kg) and two due to severe side effects.

**Table 1:** Demographic and Clinical Characteristics of Participating Children (*N* = 45)

	*M*	SD	Min	Max
Age (years)	9.53	1.70	5.91	12.77
Sex (% male)	67%			
Weight (kg)	32.7	8.41	18	64
KSADS (number of DSM symptoms present)				
Inattention symptoms	7.07	1.62	0	8
Hyperactivity-Impulsivity symptoms	5.84	2.29	2	9
ODD symptoms	2.11	2.24	0	8
SWAN Inattention				
Parent	− 14.30	6.59	− 23	4
Teacher	− 13	6.02	− 26	10
SWAN hyperactivity-impulsivity				
Parent	− 13.42	7.58	− 27	17
Teacher	− 9.49	10.62	− 26	13
CBCL Internalizing Problems (T-scores)	57.49	9.98	33	74

*CBCL* Child Behavior Checklist, *K-SADS *Kiddie–Schedule for Affective Disorders and Schizophrenia for School-Age Children–Present and Lifetime Version, *SWAN *Strength and Weakness of ADHD symptoms and Normal Behavior Rating Scale

SWAN scores may range between − 27 and 27

### Non-specific effects of methylphenidate in relation to overall effects of methylphenidate

**Fig. 2:**
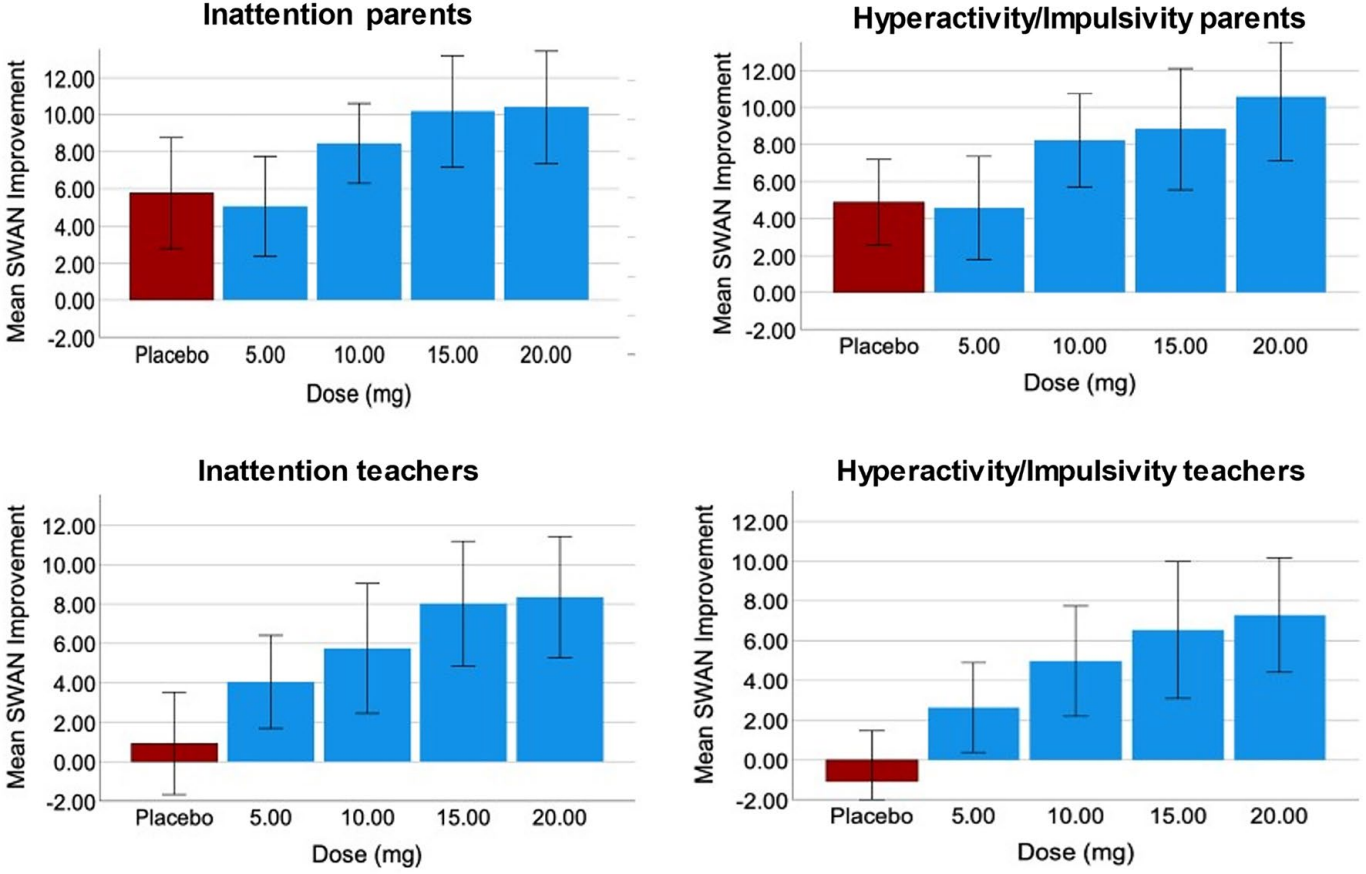
ADHD Symptom Change on the SWAN scale in Response to Methylphenidate and Placebo, Treatment as Compared to Baseline. Note: SWAN = Strength and Weakness of ADHD symptoms and Normal Behavior Rating Scale higher scores indicating more severe symptoms. Higher scores for Mean SWAN Improvement indicate larger improvements in ADHD symptoms

Figure 2 and Table 2 show the non-specific methylphenidate effects (placebo treatment versus baseline) and the overall effects (non-specific + pharmacological methylphenidate effects) for 5, 10, 15 and 20 mg methylphenidate. Parents reported significant improvement with placebo treatment and all methylphenidate doses compared to baseline on the SWAN Inattention scale and the SWAN Hyperactivity/Impulsivity scale. Visual comparison (see Fig. 2) shows that for parent-reported outcomes, non-specific effects (placebo versus baseline) are of comparable magnitude to the overall effects for 5 mg methylphenidate (non-specific + pharmacological effects) and that only for 10, 15 and 20 mg of methylphenidate, pharmacological effects add to the overall effect. Further, the pharmacological effects do not seem to exceed the non-specific effects.

**Table 2:** Effects of Placebo and Different Methylphenidate Doses versus Baseline

	Placebo (Non-specific effect)	5 mg (overall effect)	10 mg (overall effect)	15 mg (overall effect)	20 mg (overall effect)
Outcome	Coef (95%CI) Cohen’s d	Coef (95%CI) Cohen’s d	Coef (95%CI) Cohen’s d	Coef (95%CI) Cohen’s d	Coef (95%CI) Cohen’s d
SWAN inattention					
Parent	5.67*** (2.88 to 8.45) 0.64	5.07*** (2.30 to 7.83) 0.65	8.44*** (5.68 to 11.21) 1.08	10.17*** (7.38 to 12.95) 1.09	10.60*** (7.62 to 13.58) 1.32
Teacher	.72 (− 2.08 to − 3.51) 0.10	4.01** (1.23 to 6.78) 0.56	6.11*** (3.32 to 8.91) 0.67	8.61*** (5.79 to 11.42) 0.91	8.64*** (5.63 to 11.66) 1.19
SWAN hyperactivity/impulsivity					
Parent	4.88** (2.12 to 7.65) 0.57	4.58** (1.83 to 7.32) 0.54	8.22*** (5.48 to 10.97) 1.23	8.74*** (5.97 to 11.50) 1.01	10.73*** (7.77 to 13.70) 1.37
Teacher0	− 1.57 (− 4.30 to 1.16) − 0.20	2.49 (− .22 to 5.20) 0.26	4.96*** (2.23 to 7.69) 0.46	6.71*** (3.96 to 9.45) 0.73	6.85*** (3.90 to 9.80) 0.84

Note: *p < .05; **p < 0.01; ***p < 0.001 represent statistical significance compared to baseline

Teachers reported no significant improvement with placebo treatment compared to baseline on neither of the two SWAN scales. Compared to baseline, teachers reported significant improvement on the SWAN Inattention scale for all methylphenidate doses, and for 10, 15 and 20 mg of methylphenidate on the SWAN Hyperactivity/Impulsivity scale. Visual comparison shows a trivial contribution of non-specific effects to the overall effects for all active dosages on both teacher-reported SWAN scales.

### Regression to the mean effects

Parent-reported baseline SWAN Hyperactivity/Impulsivity symptoms significantly predicted parent-reported placebo effects (placebo treatment versus baseline) on this scale (*B* = 0.65, *SE* = 0.14, *p* < 0.001). Parent-reported baseline SWAN Inattention symptoms just escaped conventional levels of significance in predicting parent-reported placebo effects (placebo treatment versus baseline) on this scale (*B* = 0.28, *SE* = 0.17, *p* = 0.097). In order to understand this finding, we dichotomized parent ratings on the two SWAN scales, using medium-split analyses distinguishing between lower levels of baseline symptoms (SWAN score > median) and higher levels of baseline symptoms (SWAN score < median). We analyzed (multilevel analyses) the lower and higher baseline score group separately to determine non-specific effects in these two groups. For both the SWAN Inattention and Hyperactivity/Impulsivity scales only the group with higher baseline symptoms showed significant improvement with placebo treatment compared to baseline, see Tables 3.

**Table 3:** Non-specific effects (placebo versus baseline) for children with high and low parent reported baseline symptom of hyperactivity/ impulsivity and inattention symptoms

	Baseline symptom score	
Baseline symptom score Outcome	Low (< 14)	High (> 14)
	Coef. (95%CI) Cohen’s d	Coef. (95%CI) Cohen’s d
SWAN Inattention		
	1.92 (− 1.15 to 4.99) 0.22	9.59*** (5.11 to 14.07) 1.03
SWAN hyperactivity/impulsivity		
	2.92 (− .52 to 6.36) 0.34	7.24*** (3.22 to 11.25) 0.78

*p < .05; **p < 0.01; ***p < 0.001

SWAN ratings were dichotomized using medium-split analyses distinguishing between lower levels of baseline symptoms (SWAN score > median) and higher levels of baseline symptoms (SWAN score < median). For parent rated baseline SWAN Inattention, the median score was -14 with for the low baseline symptom group (M:− 9.87, SD: 5.62) and for the high baseline symptom group (M:− 19.55, SD: 3.03), for parent rated baseline SWAN Hyperactivity/Impulsivity − 14 with for the low baseline symptom group (M: − 8.64, SD: 6.20) and for the high baseline symptom group (M:− 19.40, SD:4.08)

### Treatment expectations

Parent-reported ‘Agreement with the diagnosis and therapy’ and ‘Treatment expectations’ did not predict higher parent-reported placebo-effects (B = − 0.15, SE = 0.52, p = 0.7747 and B = 0.45, SE = 1.76, p = 0.796, respectively). The child’s ‘Opinion on diagnosis and treatment’ and ‘Aversion towards medication’ was also not related to parent-reported placebo-effects (B = 0.15, SE = 0.73, p = 0.837 and B = 1.95, SE = 1.55, p = 0.208, respectively).

## Discussion

This study aimed to gain insight into pharmacological versus non-specific effects of methylphenidate treatment in children with ADHD using an individual randomized placebo-controlled, cross-over trial. Our results demonstrated that for parental reports of overall effect of methylphenidate on child ADHD symptoms (both inattention and hyperactivity/impulsivity symptoms), pharmacological as well as non-specific effects (i.e., significant improvement with placebo treatment) added to the overall effect of methylphenidate treatment. This result contrasts with the overall effect of methylphenidate reported by teachers, where non-specific effects did not significantly contribute to the improvement with methylphenidate treatment. The overall effect (pharmacological + non-specific effects) of the different methylphenidate dosages on all outcomes seems smaller for the teacher compared to parent ratings. However, the difference between the effects observed by teachers and parents may be fully explained by the addition of non-specific effects for parent-rated changes in ADHD symptoms. Further, we found that for parent-rated hyperactivity-impulsivity symptoms, higher baseline symptoms predicted larger non-specific effects under placebo treatment. A similar finding was obtained for parent-rated inattention, although this effect escaped conventional levels of significance. Finally, parents and child’s treatment expectations were not related to the non-specific effects under placebo treatment.

Our finding that only parents reported significant improvement in ADHD symptoms with placebo treatment, while teachers did not, aligns with the literature showing low agreement between parent and teacher reports of ADHD symptoms [6, 26–28]. The effect sizes observed for parent reports on the SWAN (improvement inattention: SMD: 0.64, hyperactivity/impulsivity: SMD: 0.57) are consistent with effect sizes reported in the meta-analysis by Faraone and colleagues (overall ADHD symptom improvement on other ADHD ratings scales that are typically used: SMD: 0.43) [6], indicating similar levels of placebo response. In contrast, our study shows no improvement in teacher ratings under placebo (improvement inattention: SMD: 0.10, hyperactivity/impulsivity: SMD: − 0.20), whereas Faraone et al. found some improvement (overall ADHD symptom improvement: SMD: 0.36), albeit effect sizes of the teacher reports were smaller than those reported by parents. This discrepancy between parents and teachers aligns with the trend observed in the literature that teacher ratings typically show lower placebo responses compared to parent ratings. This pattern is further supported by the study by Fageera et al. [23] that demonstrated greater improvement reported by parents compared to teachers with placebo treatment.

There are several potential explanations for the larger non-specific effects in parent-reported as compared to teacher-reported ADHD symptoms. One explanation is that the decision to start methylphenidate treatment is most often a co-decision between parents and clinicians, in which teachers are rarely involved. This may influence non-specific effects in two ways. First, the active decision to start methylphenidate treatment, might drive parents' desire and expectation for symptoms to improve, which are known modulators of improvement under placebo treatment [9, 29]. Second, regression to the mean effects might contribute to a larger extent to parent reported improvements. Parents may have sought treatment for their child’s ADHD symptoms at a point in time that they rate their child’s symptoms as most severe, which might be different from the symptoms perceived in the school setting, as ADHD symptoms are known to fluctuate between settings and moments in time [30, 31]. The improvements reported by the teachers will then be closer to the child’s long-term average and will therefore be less defined by regression to the mean, resulting in smaller improvement with placebo. Another potential explanation for difference between parent and teacher reports is that this study used the SWAN rating scale to assess the full range of behavior underlying symptoms of ADHD, while previous studies have used ADHD rating scales that provide assessment of just problem behaviors (weaknesses) and truncate ratings of strengths which may result in different outcomes for treatment effects.

Our findings should be viewed with some limitations in mind. First, whilst our study approach allowed to compare methylphenidate pharmacological and non-specific effects, as well as to estimate regression to the mean effects, different study designs should be used in order to complement the understanding of the regression to the mean effect and separate such effects from other non-specific effects, such as placebo effects, spontaneous improvement, patient and /or observer bias and other factors. For example, a study with double baseline measurement [32–34] might allow determination of an individual’s average symptom score prior to titration, which would allow more accurate assessment of the pharmacological methylphenidate effects because a single assessment of ADHD symptom severity may be more vulnerable to yield extreme scores compared to repeated assessments. Second, the improvement with placebo treatment made it possible to estimate regression to the mean effects. Nevertheless, an overall larger effect for active methylphenidate treatment in children with higher baseline symptoms, might not only be explained by regression to the mean, but also by larger pharmacological effects for those with more severe symptoms, leading to a statistical skewed distribution of pharmacological effects [36]. Regression to the mean effects should thus be avoided in future studies that investigate pharmacological effects for example when investigating predictors of pharmacological response. Third, we were not able to use the existing mathematical methods to estimate the magnitude of regression to the mean [32–34]. To be able to estimate the magnitude of regression to the mean, ADHD symptoms should be used both as an outcome measure as well as a selection criterium. This would allow the cut-off point for selection to be used in the mathematical model [11]. In our study we combined two measures for participant inclusion (K-SADS and DBDRS) and used a different measure to assess outcomes (SWAN), to avoid regression to the mean effects [13]. Fourth, we found no relation between treatment expectations and nonspecific effects, which may confirm our suggestion that the non-specific effects are related to regression to the mean effects. However, to accurately assess treatment expectations, it is essential to use validated questionnaires, such as the Credibility and Expectancy Questionnaire (CEQ) [37]. We recommend that future studies include such measures to provide a more comprehensive understanding of the influence of treatment expectations on nonspecific medication effects. Finally, the current study is a post-hoc analysis on the data of an RCT and was not initially powered on the outcomes presented here, which might have resulted in a lack of power. The predictive effect of parent-rated baseline inattention symptoms on the non-specific methylphenidate effects on this measure just escaped conventional levels of significance, a finding that might have turned into a significant finding with a larger sample size. Despite these limitations, our study is to the best of our knowledge, the first to explore regression to the mean as a contributing factor to non-specific and overall methylphenidate effects.

Taken together, our study shows that when parents report beneficial effects of methylphenidate on ADHD symptoms, this result is partly determined by non-specific effects. Such non-specific effects are not observed in teacher reports. These findings suggest two things. First, adding placebo treatment to the titration procedure in clinical practice might provide more insight into the extent that non-specific effects carry the observed beneficial effects of active doses of methylphenidate. Non-specific effects are not necessarily negative; however, different non-specific effects ask for a different clinical approach. Regression to the mean is a non-specific effect that should be avoided in the evaluation of methylphenidate effectiveness. It can lead to the false impression that treatment is effective, possibly leading to long term use of methylphenidate with the risk of exposure to side effects, but without the methylphenidate pharmacological benefits. In order to avoid regression to the mean effects, a double baseline measurement [33–35] can be used to determine an individual’s average symptom score prior to titration. For other non-specific effects, such as placebo effects, there is international consensus to aim at maximizing these effects by for example training health professionals in patient-clinician communication [9, 35]. Second, our findings stress the importance of including teachers as informants when interpreting the effects of methylphenidate in clinical practice. Thus, parent and teacher reports may not be interchangeable in the evaluation of pharmacological and/or placebo effects and we support the advice that optimal titration should include teachers reports to evaluate treatment effects [36, 38–40]. Further, the severity of parent-rated baseline ADHD symptoms may influence the effect of the placebo response, suggesting that placebo-controlled treatment might be particularly important for those with high symptom counts. However, we note that up until now, despite advantages shown in research PCT is not recommended in international clinical guidelines and might not be readily available in clinical practice due to the required medication kits and software for systematic registration of symptoms and side effects.

Additionally, our study did not further analyze the overall and pharmacological effects for MPH doses. If there is an overall larger effect for children with higher baseline symptom count with pharmacological treatment this might be explained by regression to the mean but also by a pharmacological larger effect for those who have more severe baseline symptoms. This means that children with more severe ADHD symptoms not only have a greater potential for measurable improvement (including regression to the mean) but may also experience a more pronounced therapeutic effect from methylphenidate, further contributing to the observed improvements [41]. For the latter symptom severity can be a cause for a skewed distribution of medication effects causing a statistical skewed distribution of pharmacological effects [42].

However, future studies may need to replicate our findings, and such studies need to use a double baseline measurement [36, 38–40] in order to determine an individual’s average symptom score prior to titration. Taken together, our findings stress the importance of assessing non-specific effects in MPH treatment outcomes in clinical and research settings.

## Supplementary Information

Below is the link to the electronic supplementary material.

Supplementary file1 (DOCX 33 KB)

## Data Availability

Deidentified individual participant data (including data dictionaries) will be made available, upon publication to researchers who provide a methodologically sound proposal for use in achieving the goals of the approved proposal. Proposals should be submitted to k.vertessen@vu.nl.
