# Supplementary material for: Placebo-related improvement with methylphenidate treatment in children with ADHD
Source: Eur Child Adolesc Psychiatry. 2024 Aug 10;34(3):1161–70. doi: 10.1007/s00787-024-02550-3 (PMC11909048; doi:10.1007/s00787-024-02550-3)
Supplement: Supplementary file 1 — Supplementary file1 (DOCX 33 KB) [file 787_2024_2550_MOESM1_ESM.docx]

**Supplement: ­­Placebo-related improvement with methylphenidate treatment in children with ADHD**

1. **Custom-made questionnaires**

3.1 Agreement with the therapy……………………………………………………...…p2

3.2 Treatment expectations………………………………………………………..……p2

3.3 Opinion on diagnosis and treatment………………………………………….….…p2

3.4 Aversion towards medication…………………………...……………………….…p3

1. **Custom-made questionnaires**

3.1 Agreement with the diagnosis and therapy

Items were scores on a 5-point scale: 1=not at all, 2=not entirely, 3=neutral, 4=a little, 5= completely. The total score of all questions combined was used, Cronbach’s Alpha for parent = .74 and teacher = .82.

Questions used were:

*Do you support the ADHD diagnosis made?*

*Do you support the general approach to treatment?*

*Do you support the treatment with medication?*

*Do you have confidence in the method by which the dose of the drugs will be determined?*

3.2 Treatment expectations

Items were scores on a 5-point scale: 1=not at all, 2=not entirely, 3=neutral, 4=a little, 5= completely. The total score was used.

Question used was:

*How much do you expect ADHD symptoms to improve?*

3.3 Opinion on diagnosis and treatment

Items were scores on a 3 point scale: 1=no , 2=a little , 3=yes. The total score of all questions combined was used, Cronbach’s Alpha = .70

Questions used were:

*- Do you dislike having ADHD?*

*- Are you ashamed of your ADHD diagnosis?*

*- Are you ashamed of your medications?*

3.4 Aversion towards medication

Items were scores on a 3 point scale: 1=no , 2=a little , 3=yes. The total score was used.

Questions used were:

*- Do you dislike taking medication?*
